# Medicaid claims alone have high sensitivity but low specificity in identifying child abuse and neglect

**DOI:** 10.3389/fped.2025.1698582

**Published:** 2026-02-02

**Authors:** Jessica Pac, Sherry Glied, Renata Howland, Jane Waldfogel

**Affiliations:** 1 Sandra Rosenbaum School of Social Work, University of Wisconsin-Madison, Madison, WI, United States; 2 New York University Robert F. Wagner Graduate School of Public Service, New York, NY, United States; 3 Department of Population Health, New York University Grossman School of Medicine, New York, NY, United States; 4 Columbia University School of Social Work, New York, NY, United States

**Keywords:** child abuse and neglect (CAN), child abuse prevention, child victimization, Medicaid claims data, predictive risk model

## Abstract

**Objectives:**

Decision support tools, such as predictive risk models (PRMs), may assist medical personnel identify child abuse and neglect (CAN). However, no prior work has established whether Medicaid claims alone are sufficient for accurate prediction. We developed a Medicaid claims-based PRM to predict CAN, validating against medical encounters and clinical diagnoses that precede and follow a CAN-related diagnosis code.

**Methods:**

We conducted a case–control study of Medicaid-enrolled children born in New York State between 2006 and 2008. Children with a CAN indicator in the first 10 years of life (case) were matched to children without (control) based on sex, race/ethnicity, county, low birthweight (<2,500 g), birth year, and months enrolled in Medicaid. Outcomes in 1 year included medical encounters, ED visits by injury intent, number of providers, and expenditures, as well as death within 5 years.

**Results:**

Of the 50,515 children in the matched sample, 13,109 (26%) were Black, 24,298 (48%) were female, 31,917 (63%) resided in New York City, and by design, 13,429 (26.5%) were diagnosed with CAN before age 10. The final PRM achieved an area under the receiver operating curve of 62%, an accuracy of 75%, a sensitivity of 98.5%, and a specificity of 9.5%. Children with a CAN indicator and those with a high probability of CAN had more high-expenditure medical encounters, particularly injury-related ED visits, compared with those without in the year following the index date.

**Conclusions:**

This claims-based tool demonstrated high sensitivity, identifying nearly all children with a CAN indicator; however, it had very low specificity. Future work should link Medicaid claims to child protective services reports to increase predictive accuracy.

## Introduction

Child abuse and neglect (CAN) is one of the nation's most pressing public health epidemics. In 2022, medical personnel submitted reports to child protective services (CPS) on behalf of 11.2% (843,000) of the 7.5 million children reported, primarily for neglect (66.2%) and physical abuse (19.5%) (1). Many physical indications of CAN, such as sentinel bruising, are difficult to distinguish from accidental injuries or conditions. In the emergency department (ED), identification is further hampered by the lack of historical and contextual information about the child and their caregiver(s), as clinicians are limited to the information available in medical records. However, early and accurate identification of CAN is imperative. An estimated 20%–50% of fatal CAN victims had at least one previous medical encounter at which the signs of CAN were present but not diagnosed or reported to CPS (2–6). In response, many US medical systems are piloting or implementing decision support tools to provide additional information to medical personnel when faced with the complex, high-stakes decision of whether to notify CPS (7–10).

In this paper, we develop a predictive risk model (PRM) to identify CAN exclusively from Medicaid claims and enrollment data. Whereas previous work demonstrated that ED encounters can be used to predict CAN when health records are matched to administrative child welfare data, decision tools requiring external data linkages cannot be used in practice due to privacy restrictions. Therefore, the aim of this study is to determine whether unlinked administrative medical records, in this case Medicaid claims, can be used to predict CAN in support of clinician decision-making (11). Specifically, our model identifies the pattern of injuries or conditions that led to the first, formal indication of CAN in Medicaid claims. These indications may be confirmed by medical providers or by CPS. The latter is a function of both objective harm and the medical personnel's propensity to notify CPS, whereas the former can be interpreted as a more objective measure of harm.

Our secondary aim is to document the pattern of injuries and conditions that follow the first indication of CAN in Medicaid claims. Comparing health and medical use outcomes within one year yields the unadjusted “effect” of a CAN indication in medical records. Relative to children at a high risk of CAN, comparing post-visit outcomes yields insight into the consequence of a “missed” diagnosis.

Unlike CPS referrals that generally lack information on contextual and historical factors preceding the CPS encounter, Medicaid claims capture a rich, detailed history of the physical and often social risk factors for CAN. Previous studies have highlighted the potential of Medicaid claims for predicting CAN by using diagnosis and procedure codes to construct the following predictors: types and locations of injuries, the nature and number of previous encounters overall and by specific providers (e.g., emergency department physicians), specific diagnoses, such as traumatic brain injuries and sentinel bruising, and patterns of medical use that are associated with CAN. We leverage Medicaid claims to predict CAN for these reasons. In addition, (1) most children who encounter the child welfare system are enrolled in Medicaid at the time of their encounter (70%–80%) (12, 13) (2) the data are longitudinal and standardized across clinical contexts, and (3) concerns with reporting bias—which plague administrative child welfare data—are minimized (7, 11).

## Materials and methods

### Data and study design

Our sample consists of 365,598 children with Medicaid-funded births in New York State between 2006 and 2008. Medicaid claims were accessed through the Health Evaluation and Analytics Lab (HEAL) at NYU and include all fee-for-service claims and managed care encounters for individuals insured by Medicaid in NYS. We identify children with any indication of suspected or confirmed CAN (“cases”) according to the *International Classification of Diseases Clinical Modification* codes, including ICD-9-CM (9955 series, V6121, V7181, V715) and ICD-10-CM (T74, T76, Z69010, Z0472, Z0471, and Z0441) codes. We refer to these codes throughout as CAN indicators, and select the first CAN indicator to appear in Medicaid claims as the index event (14). Children in the control group did not have a CAN indicator at any point in their first ten years of life.

We constructed a case–control sample using a 3-to-1 greedy propensity score matching (with replacement) based on children's residential location (county at time of birth), birth year and month, race/ethnicity (white, Black, Asian or Pacific/Islander, Hispanic, other races, and unknown), sex (male, female), low birthweight (<2,500 g at birth), SSI/TANF receipt, and months of Medicaid enrollment over the 10-year period. Our final analytic sample consisted of 50,515 children, including 26.58% (*n* = 13,429) case children with a CAN indicator and 73.42% (*n* = 37,086) control children without. Henceforth, the term “CAN” refers to the first indication of suspected or confirmed CAN in Medicaid claims. This study was reviewed by the Institutional Review Board at New York University and was considered exempt as a secondary analysis of existing data.

### Statistical analysis

In the first phase of our analysis, we applied machine learning to identify the patterns of diagnoses and medical encounters that predict CAN. We began with a list of 292 diagnosis codes identified in previous retrospective case note analyses as detailed in Supplementary Material (11, 15). After randomly partitioning our sample into a 70/30 train/test set, we used gradient boosted trees to predict CAN, an algorithm with increased accuracy in predicting rare outcomes (16). We employed fivefold cross-validation using out-of-bag estimates of error to select hyperparameters, including tree number, complexity, and learning rate. We trained models to optimize the area under the receiver operating curve (AUC). We considered models with all candidate predictors, as well as a subset without the medical encounter predictors.

In the second phase of our analysis, we tested for differences across quartiles of predicted CAN risk using chi-square tests and by estimating relative risks. We exploited the longitudinal nature of the data to validate our model by comparing the rate of subsequent outcomes and medical encounters across children with CAN versus quartiles of predicted CAN risk in the following year, except for death, which we observe within 5 years (17–19). We selected a set of outcomes that either reflected an omission of preventative care, potentially excessive emergent care, and any injuries related to these visits. We additionally constructed a new outcome—a high-risk CAN indicator—based on the diagnosis codes identified in phase 1 (shown in Supplementary eTable 2). To account for potential confounding due to changes in Medicaid enrollment, we created average rates by months enrolled. All data manipulation and propensity score matching were conducted in SAS (SAS Institute, Cary, NC). All models were run in R.

## Results

### Sample characteristics

Table 1 presents the characteristics of the full sample (columns 1 and 2), children with a CAN indicator (case, columns 3 and 4), those without (control, columns 5 and 6), and the control group disaggregated by quartile of CAN risk (columns 7 through 14). The full sample of case and control children are similar on matched characteristics, but the demographic characteristics of children within the control group vary substantially when we disaggregate the control sample by CAN risk quartile.

**Table 1:** Sample characteristics for case and controls, and by predicted risk of maltreatment.

Variables	No. (%)			Control (*n* = 37,086)			
	Matched sample (*n* = 50,515)	Case (*n* = 13,429)	Control (*n* = 37,086)	Bottom 25% (*n* = 9,271)	Bottom 50% (*n* = 18,543)	Top 50% (*n* = 18,543)	Top 25% (*n* = 9,272)
Age at time of visit, years							
<1	11,785 (23.33)	3,409 (25.39)	8,376 (22.59)	1,767 (19.06)	3,253 (17.54)	5,123 (27.63)	3,017 (32.54)
2–5	14,651 (29.00)	3,911 (29.12)	10,740 (28.96)	2,430 (26.21)	5,074 (27.36)	5,666 (30.56)	2,804 (30.24)
5–10	24,079 (47.67)	6,109 (45.49)	17,970 (48.45)	5,074 (54.73)	10,216 (55.09)	7,754 (41.82)	3,451 (37.22)
Race/ethnicity							
Unknown	5,045 (9.99)	1,339 (9.97)	3,706 (9.99)	986 (10.64)	1,971 (10.63)	1,735 (9.36)	804 (8.67)
White	13,867 (27.45)	4,108 (30.59)	9,759 (26.31)	1,985 (21.41)	4,125 (22.25)	5,634 (30.38)	3,050 (32.89)
Black	13,109 (25.95)	3,348 (24.93)	9,761 (26.32)	2,295 (24.75)	4,707 (25.38)	5,054 (27.26)	2,565 (27.66)
Asian	1,979 (3.92)	499 (3.72)	1,480 (3.99)	636 (6.86)	1,036 (5.59)	444 (2.39)	175 (1.89)
Other	1,372 (2.72)	430 (3.20)	942 (2.54)	290 (3.13)	517 (2.79)	425 (2.29)	191 (2.06)
Hispanic	15,143 (29.98)	3,705 (27.59)	11,438 (30.84)	3,079 (33.21)	6,187 (33.37)	5,251 (28.32)	2,487 (26.82)
Sex							
Female	24,298 (48.10)	6,514 (48.51)	17,784 (47.95)	4,709 (50.79)	9,204 (49.64)	8,580 (46.27)	4,132 (44.56)
Male	26,217 (51.90)	6,915 (51.49)	19,302 (52.05)	4,562 (49.21)	9,339 (50.36)	9,963 (53.73)	5,140 (55.44)
SSI/TANF							
No	41,551 (82.25)	10,819 (80.56)	30,732 (82.87)	8,053 (86.86)	15,866 (85.56)	14,866 (80.17)	7,378 (79.57)
Yes	8,964 (17.75)	2,610 (19.44)	6,354 (17.13)	1,218 (13.14)	2,677 (14.44)	3,677 (19.83)	1,894 (20.43)
Low birthweight							
No	46,537 (92.13)	12,255 (91.26)	34,282 (92.44)	8,646 (93.26)	17,234 (92.94)	17,048 (91.94)	8,519 (91.88)
Yes	3,978 (7.87)	1,174 (8.74)	2,804 (7.56)	625 (6.74)	1,309 (7.06)	1,495 (8.06)	753 (8.12)
Geography							
NYC	31,917 (63.18)	8,882 (66.14)	23,035 (62.11)	4,546 (49.03)	9,917 (53.48)	13,118 (70.74)	6,996 (75.45)
Not NYC	18,598 (36.82)	4,547 (33.86)	14,051 (37.89)	4,725 (50.97)	8,626 (46.52)	5,425 (29.26)	2,276 (24.55)
Birth year							
2006	15,047 (29.79)	4,035 (30.05)	11,012 (29.69)	2,499 (26.96)	5,210 (28.10)	5,802 (31.29)	2,965 (31.98)
2007	16,958 (33.57)	4,507 (33.56)	12,451 (33.57)	3,188 (34.39)	6,326 (34.12)	6,125 (33.03)	3,055 (32.95)
2008	18,510 (36.64)	4,887 (36.39)	13,623 (36.73)	3,584 (38.66)	7,007 (37.79)	6,616 (35.68)	3,252 (35.07)

### Predictive risk model

The final gradient boosting algorithm included the medical encounter variables and yielded an AUC of 0.62 in the validation set, meaning that the model is only moderately good at discriminating between children with and without CAN. Overall accuracy in the full sample was 0.748, considered acceptable for prediction. Evaluation metrics for both models with and without the medical encounter variables, for both the test and full dataset, are presented in Supplementary eTable 1. Our final model (column 4, Supplementary eTable 1) exhibited accuracy (precision) of 0.748, sensitivity (recall) of 0.985, and specificity of 0.095. This indicates that our model accurately identifies children who will subsequently have a CAN indicator (few false negatives), but does so by casting a wide net—flagging many children who ultimately never have a CAN indicator (false positives). Despite exhibiting moderate accuracy, the relatively low AUC (0.54) and specificity (0.095) indicate that the yield of this model is low and not particularly informative (11, 20, 21).

We find that eight medical encounter variables are the strongest predictors of CAN (shown in Supplementary eTable 2): Medicaid expenditures, number of claims, number of ED visits, number of primary care providers, number of providers, number of visits to specialists, number of vaccines, and number of well-child visits. Three of these variables, observed in the month before the index visit, were highly associated with CAN: Medicaid expenditures for the last month, number of claims in the last month, and the number of providers in the last month. This result implies that case children receive intensive medical care in the month prior to their index visit.

The top 20 diagnostic predictors from our final model are shown in Supplementary eTable 3, with exclusion codes shown in Supplementary eTable 4. The most predictive diagnoses of CAN include contusions (bruising); asthma; disorders related to short gestation and low birthweight; convulsions; unexplained failure to thrive; housing and economic circumstances; hepatitis B, C, delta, and E; poisoning; unexplained nutritional deficiencies; unexplained abnormal weight loss; scabies; traumatic brain injury; pregnancy; substance poisoning; encopresis; diabetic retinopathy; and dental caries. Of these, only four diagnostic codes appear to predict CAN within the subsequent month: disorders relating to short gestation and low birthweight, contusions, unexplained abnormal weight loss, and convulsions. As there are multiple, plausible explanations for each of these codes, in addition to CAN, we caution against considering them as independently predictive of CAN.

To further facilitate interpretation, we present SHAP (SHapley Additive exPlanations) values in Supplementary eFigure 3. The leftmost bar chart ranks predictors by their absolute SHAP value, indicating that contusions, asthma, and convulsions are the most important features for prediction. The beeswarm figure on the right shows the heterogeneity in feature contributions across individual observations. Although most SHAP values are concentrated near zero, those with non-zero contributions—asthma, disorders relating to preterm birth or low birthweight, and femur fracture—indicate that positive values are concentrated on a small subset of cases, signifying low prevalence but high conditional importance.

### Patterns of care after a visit with a CAN indicator

Table 2 presents the outcomes observed after the index visit across case and control groups. Specifically, in the year after the index visit, we compare case and control children in terms of medical encounters, ED visits, and death within 5 years. We find that case children faced a threefold risk of death in 5 years. Case children were 1.88 times more likely to have any of the high-risk CAN indicators (listed in Supplementary eTable 2) in the year following the index visit, relative to those without. On average, case children had 4.21 high-risk CAN indicators compared with 1.72 in the control group. Case children, relative to controls, were also twice as likely to have an ED encounter and over three times as likely to have an inpatient stay. Among the reasons for ED visits shown in Panel B, case children faced 3.35 times the risk of having a self-harm injury, 3.47 times the risk of an unintentional injury, 2.29 times the risk of an injury with an unknown origin, and 4.3 times the risk of having any injury-related visit. Control children had a very low prevalence of assault-related injuries (the exact number was suppressed for privacy reasons) relative to case children (8). Interestingly, case children exhibited higher rates of preventative care, with a moderately higher risk of primary care and well-child visits (1.16 and 1.17 times, respectively).

**Table 2:** Risk ratios of children's outcomes 12 months after index visit.

Variables	No. (%)		
	Case (*n* = 13,429)	Control (*n* = 37,086)	RR (95% CI)
Panel A: Outcomes			
Death (5 years)	36 (0)	33 (0)	3.01 (1.88–4.84)
Any high-risk diagnosis	4,786 (35.64)	7,036 (18.97)	1.88 (1.82–1.94)
Count of high-risk diagnosis	4.21 (23.0)	1.72 (21.0)	2.46 (1.87–2.10)
At least 1 ED visit	7,027 (52.33)	9,636 (25.98)	2.01 (1.97–2.06)
At least 1 inpatient visit	1,606 (11.96)	1,382 (3.73)	3.21 (2.99–3.44)
At least 1 primary care visit	12,514 (93.19)	29,760 (80.25)	1.16 (1.15–1.17)
At least 1 well-child visit	10,239 (76.25)	24,162 (65.15)	1.17 (1.17–1.18)
Panel B: Emergency Department visits by concern			
Self-harm	605 (4.51)	498 (1.34)	3.35 (2.98–3.77)
Unintentional injury	1,291 (9.61)	1,028 (2.77)	3.47 (3.20–3.76)
Assault	258 (1.92)	^a^	
Unknown	44 (0)	53 (0)	2.29 (1.54–3.74)
Any injury related	2,298 (17.11)	1,477 (3.98)	4.30 (4.04–4.57)

RR, risk ratio.

a Small cell sizes that prevent calculations.

The results in Table 3 compare case children (columns 1 and 2) across CAN probability quartiles estimated using PRM. Results in Panel A suggest that as CAN risk increases, comparing Q4 to Q1 for example (columns 9 and 10 to 3 and 4), the risk of adverse outcomes resembles case children (columns 1 and 2). The risk of death within 5 years was 2.83 times higher among the children in the top quartile of predicted CAN risk (Q4) relative to those below the median (columns 5 and 6). Children in Q4 exhibited more high-risk CAN diagnosis and were more likely to be admitted to the ED and inpatient. The count of high-risk diagnosis codes in the control group increased between Q1 and Q4 (with those in Q4 having 2.23 more high-risk CAN indicators in Supplementary eTable 2, on average), suggesting that the accumulation of high-risk CAN indicators could be a meaningful indicator of CAN. Like the case–control comparison shown in Table 2, children in Q4 were only modestly more likely to have at least one visit with a primary care provider; however, unlike the case–control comparison, children in Q4 were less likely to have well-child visits, suggesting that children who faced a high CAN were more likely to be observed by specialized providers and emergency physicians. The types of concerns associated with ED visits followed the anticipated pattern (Panel B), with Q4 children facing 2.02 times the risk of self-harm injuries, 1.81 times the risk of unintentional injuries, 3.47 times the risk of injuries with unknown intent, and 1.84 times the risk of any injury-related visit.

**Table 3:** Risk ratios of children's outcomes 12 months after index visit, by predicted CAN quartiles.

Variables	No. (%)					
	Case (*n* = 13,429)	Control, bottom 25% (*n* = 9,271)	Control, bottom 50% (*n* = 18,543)	Control, top 50% (*n* = 18,543)	Control, top 25% (*n* = 9,272)	Top 25% vs. bottom 25%, RR (95% CI)
Panel A: Outcomes						
Death (5 years)^b^	36 (0)	^a^	12 (0)	21 (0)	17 (0)	2.83 (1.12–7.18)
Any high-risk diagnosis	4,786 (35.64)	1,383 (14.92)	2,990 (16.12)	4,046 (21.82)	2,204 (23.77)	1.59 (1.50–1.69)
Count of high-risk diagnosis	4.21 (23.0)	1.29 (10.35)	1.21 (8.04)	2.22 (28.61)	2.89 (39.41)	2.23 (1.62–3.08)
At least 1 ED visit	7,027 (52.33)	1,845 (19.90)	3,966 (21.39)	5,670 (30.58)	3,043 (32.82)	1.65 (1.57–1.73)
At least 1 inpatient visit	1,606 (11.96)	306 (3.30)	601 (3.24)	781 (4.21)	438 (4.72)	1.43 (1.24–1.65)
At least 1 primary care visit	12,514 (93.19)	7,301 (78.75)	14,651 (79.01)	15,109 (81.48)	7,609 (82.06)	1.04 (1.03–1.06)
At least 1 well-child visit	10,239 (76.25)	6,112 (65.93)	12,184 (65.71)	11,978 (64.60)	5,954 (64.21)	0.97 (0.95–0.99)
Panel B: Emergency Department visit by concern						
Self-harm	605 (4.51)	86 (1)	200 (1.08)	298 (1.61)	174 (1.88)	2.02 (1.57–2.62)
Unintentional injury	1,291 (9.61)	183 (1.97)	402 (2.17)	626 (3.38)	332 (3.58)	1.81 (1.52–2.17)
Assault	258 (1.92)	^a^	^a^	^a^	^a^	
Unknown^b^	44 (0)	^a^	15 (0)	38 (0)	26 (0)	3.47 (1.61–8.55)
Any injury related	2,298 (17.11)	263 (2.84)	586 (3.16)	891 (4.81)	484 (5.22)	1.84 (1.59–2.13)
Panel C: Medical use patterns						
Number of claims per year						
0	0	494 (5.33)	1,033 (5.57)	971 (5.24)	477 (5.14)	0.97 (0.85–1.09)
<100	12,804 (95.35)	8,628 (93.06)	17,209 (92.81)	17,004 (91.70)	8,453 (91.17)	0.98 (0.97–0.99)
100+	625 (4.65)	149 (1.61)	301 (1.62)	568 (3.06)	342 (3.69)	2.30 (1.90–2.78)
Number of ED visits/year						
0	6,402 (47.67)	7,425 (80.09)	14,577 (78.61)	12,873 (69.42)	6,229 (67.18)	0.84 (0.82–0.85)
<5	6,566 (48.89)	1,759 (18.97)	3,798 (20.48)	5,364 (28.93)	2,863 (30.88)	1.63 (1.55–1.71)
5+	461 (3.43)	86 (0.93)	168 (0.91)	306 (1.65)	180 (1.94)	2.09 (1.62–2.70)
Number of primary care visits/year						
0	915 (6.81)	1,970 (21.25)	3,892 (20.99)	3,434 (18.52)	1,663 (17.94)	0.84 (0.80–0.90)
<5	5,733 (42.69)	4,336 (46.77)	8,730 (47.08)	8,109 (43.73)	3,866 (41.70)	0.89 (0.86–0.92)
5+	6,781 (50.50)	2,965 (31.98)	5,921 (31.93)	7,000 (37.75)	3,743 (40.37)	1.26 (1.21–1.31)
Average of providers/month	8.47	4.62	4.78	5.59	5.84	1.22
Average Medicaid costs/month	$5,636.66	$2,134.32	$2,046.38	$2,575.61	$3,035.18	$900.86
Average number of vaccines/month	7.99	10.244	9.777	7.181	6.598	−3.65

RR, risk ratio.

a Small cell sizes that prevent calculations.

b Due to small sizes in bottom 25%, we compare top 25% to bottom 50%.

We examine the patterns of medical use among case relative to control children disaggregated by risk quartile in Panel C. These results indicated that as the risk of CAN increased (moving from columns 3 and 4 to 9 and 10), medical use patterns more closely resembled case children in terms of the number of claims per year, the number of emergency department visits/year, and the number of primary care visits/year. Across all three categories of encounters, children with the highest risk of CAN were more likely to rely on emergency and specialized care and have more primary care visits per year. Children in Q4 saw on average 1.22 more providers, incurred $900.86 in additional Medicaid expenditures, and received 3.65 fewer vaccines, relative to low-risk children (quartile 1).

## Discussion

Our first aim was to determine whether PRM can predict CAN exclusively from Medicaid claims. We trained a gradient boosted trees machine learning algorithm to predict CAN in Medicaid-enrolled children ages 10 and younger. While our final model exhibited a high sensitivity (98.5%) and moderate accuracy (74.8%), the relatively low specificity (9.5%) and AUC (62.2%) suggest that additional data (e.g., clinical notes, case narratives) and research are needed to improve model discrimination to supplement clinical decision-making in practice. An unwarranted CPS report could bring unnecessary harm to families and children who have a low CAN risk, implying that medical personnel should consider alternative decision support tools (8, 22, 23). Yet, as unsubstantiated CPS reports are highly predictive of later substantiated investigations (24), fatal injuries (25, 26), and ED use (27), the benefits of early intervention for those with a high CAN risk should be carefully weighed against the potential costs of a false positive.

For comparison, the Allegheny Family Screening Tool (AFST)—a PRM trained to predict removal and placement into foster care using CPS data linked to a range of social services data—exhibited a considerably higher AUC of 0.78, emphasizing the potential of data-driven decision support tools (11, 20).

Our second aim was to examine the pattern of diagnosis and medical encounters following the index visit, defined as the medical visit at which the first indication of CAN appears in children's Medicaid claims. Our results indicate that in the year following the index visit, case children had substantially higher risk of adverse outcomes and more high-expenditure medical encounters, such as ED visits and inpatient admissions, as well as slightly more low-expenditure, preventative care visits. Case children exhibited higher rates of ED visits for reasons related to self-harm, unintentional injury, unknown causes, and any injury encounter. They had nearly double the number of monthly medical encounters relative to low-risk children, implying that abused and neglected children should be closely monitored by medical personnel, regardless of whether or not the child is removed from their home.

Although only a small fraction of case children died within 5 years, the risk of death was threefold that of control children. Case children also visited the ED for injury-related concerns at over four times the rate of those without for reasons related to assault, self-harm, and accidental injuries. This finding reiterates previous research revealing that physical abuse injuries are screened and identified at higher rates than injuries or conditions related to neglect, which—while similarly consequential to children's longevity and wellbeing—lack the physical indicators required for unbiased, early diagnosis (7). In addition, these results imply that case children were either not reported to CPS or that CPS interventions did not match the level of risk experienced by the child. Without CPS data, we are unable to rule out either option.

Among control children, those with a high predicted probability of CAN exhibited a patterns similar to case children in terms of high-expenditure ED and inpatient encounters, and fewer low-expenditure, preventative encounters, such as well-child visits and vaccine administrations.

### Limitations and ethical considerations

Although our findings offer novel insight into the potential of a Medicaid claim-based screening tool for CAN, our study is not without limitations. First, we were unable to observe CPS reports, investigations, and removals, which prevented us from validating the algorithm as a screening tool. Without additional information on encounters with CPS and later trajectories, we cannot estimate the costs or benefits of formal intervention in terms of children's objective wellbeing. Relatedly, deaths are vastly underreported in Medicaid claims, further limiting validation (28).

Second, our findings are not generalizable to all children because our sample was limited to Medicaid-enrolled children in a single state, New York, for which Medicaid eligibility and enrollment differ from other states. Third, our results should not be interpreted as causal as our approach was implicitly focused on prediction, rather than identifying causal pathways.

Despite the many benefits of using Medicaid claims to predict child maltreatment for the purposes of facilitating early—and ostensibly less severe—intervention, there are two additional ethical considerations worth noting. First, a baseline concern with implementing algorithmic prediction models using a selected sample, as is the case of our reliance on Medicaid claims, is that the resulting predictions may capture variation due to poverty, rather than maltreatment risk, thus reinforcing existing disparities. However, as our model was trained to predict maltreatment identification within the Medicaid system, it is plausibly less biased than a model that predicts CPS reporting, because parents voluntarily seek medical care, whereas CPS reporting is involuntary and subject to race- and class-based bias. Relatedly, a second concern is whether Medicaid claims should be repurposed to identify children who face a high risk of maltreatment, given that the data were not collected for this purpose. The objective of our analysis was to test whether stand-alone claims can reliably predict maltreatment, with particular attention to minimizing type I and type II errors. We conclude that the benefits of using claims alone do not outweigh the accuracy-related costs.

## Conclusions

While many county and state CPS agencies use PRM and other decision support tools to aid social workers in high-stakes screening and removal decisions, such as the pathbreaking AFST, tools to support medical personnel in medical settings primarily consist of EHR-based screeners and triggers, training programs, and child abuse pediatric teams (7, 8, 10, 11, 29). Although several screening tools demonstrate remarkably high predictive accuracy for physical abuse injuries, few screen children for all forms of abuse and neglect, and the use of PRMs in medical settings is limited in practice (7–10, 22, 23, 30–32). The results of our analysis suggest that PRMs based entirely upon Medicaid claims exhibits low-to-moderate accuracy and should not be used as a substitute for human intuition.

That said, our analysis yields two findings regarding the patterns of outcomes after maltreatment. First, in the year following the first indication of CAN in medical records, children are more likely to have subsequent high-risk CAN indicators (Supplementary eTable 2) and ED encounters for any form of injury, self-harm or otherwise. Second, the diagnosis and medical use patterns of control children with a high CAN risk resembled those of case children, implying that screening tools could rely on a relatively short list of diagnostic codes and encounters to facilitate earlier intervention for at-risk children. Rather than waiting until CAN is confirmed to generate a CPS referral, earlier identification of CAN risk would enable medical providers to address children's needs immediately through targeted interventions, referrals to public assistance such as nutritional or income support programs, or family support services that are less intensive than CPS.

## Data Availability

The data analyzed in this study are subject to the following licenses/restrictions: the HEAL data are restricted and only accessible by approved researchers. Requests to access these datasets should be directed to John Billings, john.billings@nyu.edu.
