# Supplementary material for: Medicaid claims alone have high sensitivity but low specificity in identifying child abuse and neglect
Source: Front Pediatr. 2026 Feb 2;13:1698582. doi: 10.3389/fped.2025.1698582 (PMC12907437; doi:10.3389/fped.2025.1698582)
Supplement: Supplementary file 1 [file Datasheet1.docx]

**Online Appendix**

**eMethods1.** Description of candidate predictors

In the first phase, we use machine learning methods to identify the pattern of diagnosis and medical encounters that predict CAN. Rather than use all diseases and conditions, we turned to a prior study that used retrospective case note analysis to identify the ICD-9-CM diagnosis and exclusion codes that commonly preceded a confirmed CAN diagnosis, then created a crosswalk to ICD-10-CM.^1^ We determined whether each diagnostic code appeared in the month prior to the index visit or at any point in time prior to the previous month. For the control group, the index visit date was randomly imputed based on the distribution of enrollment dates and all outcomes were measured in the 12 months following start date. Since some diagnostic codes could result in injuries that resemble maltreatment but are attributable to other causes, such as motor vehicle accidents and malnourishment due to cancer, we follow the same study to exclude these and other conditions unrelated to CAN, shown in eTable4. The resulting list of 292 “feature” codes captures an array of injuries and conditions proximally related to physical abuse, sexual abuse, and neglect, including traumatic brain injuries, sexually transmitted diseases and other communicable diseases, dental caries, injuries due to inadequate supervision (near drowning in the bathtub/pool, burns, poisoning, etc.), and indicators of physical neglect (e.g. unexplained failure to thrive, body lice, scabies, etc.). We also include a set of variables that summarize medical encounters in the month before the reference visit and in the total period prior to the previous month as well as medical use summary measures including number of primary care providers and specialists, Medicaid expenditures, and overall number of providers.

| **eTable1. Gradient boosted trees, evaluation metrics** | | | | |
| --- | --- | --- | --- | --- |
|  | **Test** | **Full** | **Test** | **Full** |
| Diagnostic codes | x | x | x | x |
| Medical use codes |  |  | x | x |
| Accuracy | 0.738 | 0.736 | 0.739 | 0.748 |
| 95% CI | (0.731, 0.7451) | (0.7316, 0.7393) | (0.7319, 0.7459) | (0.7446, 0.7522) |
| P-Value [Acc > NIR] | 0.482 | 0.252 | 0.388 | 0.000 |
| Kappa | 0.007 | 0.012 | 0.064 | 0.111 |
| Mcnemar's Test P-Value | <2e-16 | <2e-16 | <2e-16 | < 2.2e-16 |
| Sensitivity | 0.998 | 0.998 | 0.977 | 0.985 |
| Specificity | 0.007 | 0.010 | 0.069 | 0.095 |
| PPV | 0.739 | 0.736 | 0.747 | 0.750 |
| NPV | 0.528 | 0.672 | 0.515 | 0.696 |
| Prevalence | 0.738 | 0.734 | 0.738 | 0.734 |
| Detection rate | 0.736 | 0.733 | 0.721 | 0.723 |
| Detection prevalence | 0.997 | 0.996 | 0.965 | 0.964 |
| Balanced accuracy | 0.502 | 0.504 | 0.523 | 0.540 |
| F1 | 0.849 | 0.847 | 0.847 | 0.852 |
|  |  |  |  |  |

| **eTable3. Exclusion codes** | |  |
| --- | --- | --- |
| **Exclusion category** | **ICD-9 Code** | **Definition** |
| Nutritional (EN) | 9 | Infections of the gastrointestinal tract |
| Nutritional (EN) | 42 | HIV disease |
| Nutritional (EN) | 271.3 | Lactose intolerance |
| Nutritional (EN) | 277.0 | Cystic fibrosis |
| Nutritional (EN) | 431 | Intracerebral hemorrhage |
| Nutritional (EN) | 530.81 | Gastroesophageal reflux |
| Nutritional (EN) | 577.8 | Pancreatic insufficiency |
| Nutritional (EN) | 588.8 | Renal tubular acidosis |
| Nutritional (EN) | 593.9 | Chronic renal insufficiency |
| Nutritional (EN) | 599.0 | Urinary tract infection |
| Nutritional (EN) | 750.5 | Pyloric stenosis |
| Nutritional (EN) | 751.3 | Hirschsprung's disease |
| Nutritional (EN) | 760.71 | Fetal alcohol syndrome |
| Nutritional (EN) | 767.0 | Birth trauma: subdural/cerebral hemorrhage |
| Nutritional (EN) | 770.7 | Bronchopulmonary dysplasia |
| Nutritional (EN) | 010.0–018.9 | Tuberculosis |
| Nutritional (EN) | 070.00–070.9 | Viral hepatitis |
| Nutritional (EN) | 140.0–208.91 | Malignancy |
| Nutritional (EN) | 243–244.9 | Hypothyroidism |
| Nutritional (EN) | 250.00–250.93 | Diabetes mellitus |
| Nutritional (EN) | 252.0–252.9 | Parathyroid disorders |
| Nutritional (EN) | 253.0–253.9 | Disorders of the pituitary gland and its hypothalamic control |
| Nutritional (EN) | 270.0–275.9 | Inborn errors of metabolism |
| Nutritional (EN) | 317–319 | Mental retardation |
| Nutritional (EN) | 330.0–344.42 | Neurologic hereditary, degenerative, and other disorders |
| Nutritional (EN) | 446.0–446.7 | Polyarteritis nodosa and allied conditions |
| Nutritional (EN) | 493.00–493.92 | Asthma |
| Nutritional (EN) | 555.0–558.9 | Inflammatory bowel disease |
| Nutritional (EN) | 571.0–571.9 | Cirrhosis |
| Nutritional (EN) | 575.0–576.9 | Biliary disease |
| Nutritional (EN) | 579.0–579.9 | Intestinal malabsorption |
| Nutritional (EN) | 710.0–710.9 | Diffuse diseases of connective tissue |
| Nutritional (EN) | 714.0–714.9 | Rheumatoid arthritis and other inflammatory polyarthropathies |
| Nutritional (EN) | 745.0–747.9 | Cardiac disease, congenital |
| Nutritional (EN) | 749.00–749.25 | Cleft palate/cleft lip |
| Nutritional (EN) | 758.0–758.9 | Chromosomal abnormalities |
| Nutritional (EN) | 771.0–771.89 | Perinatal infections |
| Nutritional (EN) | 772.10–772.14 | Intraventricular hemorrhage |
| Nutritional (EN) | 852.00–853.19 | Subarachnoid, subdural, extradural, other/unspecified, hemorrhage following injury |
| Nutritional (EN) | 984.0–984.9 | Lead poisoning |
| Motor vehicle (MV) | E810-E813 and E815-E819 unless 0.6 or 0.7 |  |
|  |  |  |

| **eTable4. Medical encounter predictors** | |
| --- | --- |
| Medicaid costs, last 30 days |  |
| Number of claims |  |
| Number of claims, last 30 days |  |
| Number of ED visits |  |
| Number of primary care providers |  |
| Number of providers |  |
| Number of providers, last 30 days |  |
| Number of specialties |  |
| Number of vaccines |  |
| Number of well child visits |  |
| Total Medicaid costs |  |

**eFigure1. Sample construction**

**Predictors**

**Case child**

**Control child**

3-1 matching on child’s county at birth, age in months, race/ethnicity, sex, low birthweight status, SSI/TANF receipt status, months of Medicaid enrollment.

*12 mo.*

*5 years*

*Birth - 1 mo. before*

*1 mo.*

Birth to 10 years

Death

**Outcomes**

First CAN indication in claims (index visit)

292 variables capturing diagnosis codes and healthcare utilization*

(1) Any high-risk diagnosis; (2) having 1+ visit by type; (3) ED visit for injuries

Death

No CAN indication in claims

292 variables capturing diagnosis codes and healthcare utilization*

(1) Any high-risk diagnosis; (2) having 1+ visit by type; (3) ED visit for injuries

*See eTables2-4 for list of predictors

**eFigure2. Analytic workflow**

70% of full sample, split into 5 folds

Test model

Identify key parameters

External validation

Outcomes 12 months after index visit, death in 5 yr.

Train

Test

Train

Train

Train

Train

Test

Train

Train

Train

Train

Test

Train

Train

Train

Train

Test

Train

Train

Train

Train

Test

Train

Train

Train

30% of full sample

Full sample

**Phase 1: Model training**

**Phase 2: External validation**

**eFigure3. SHAP values**


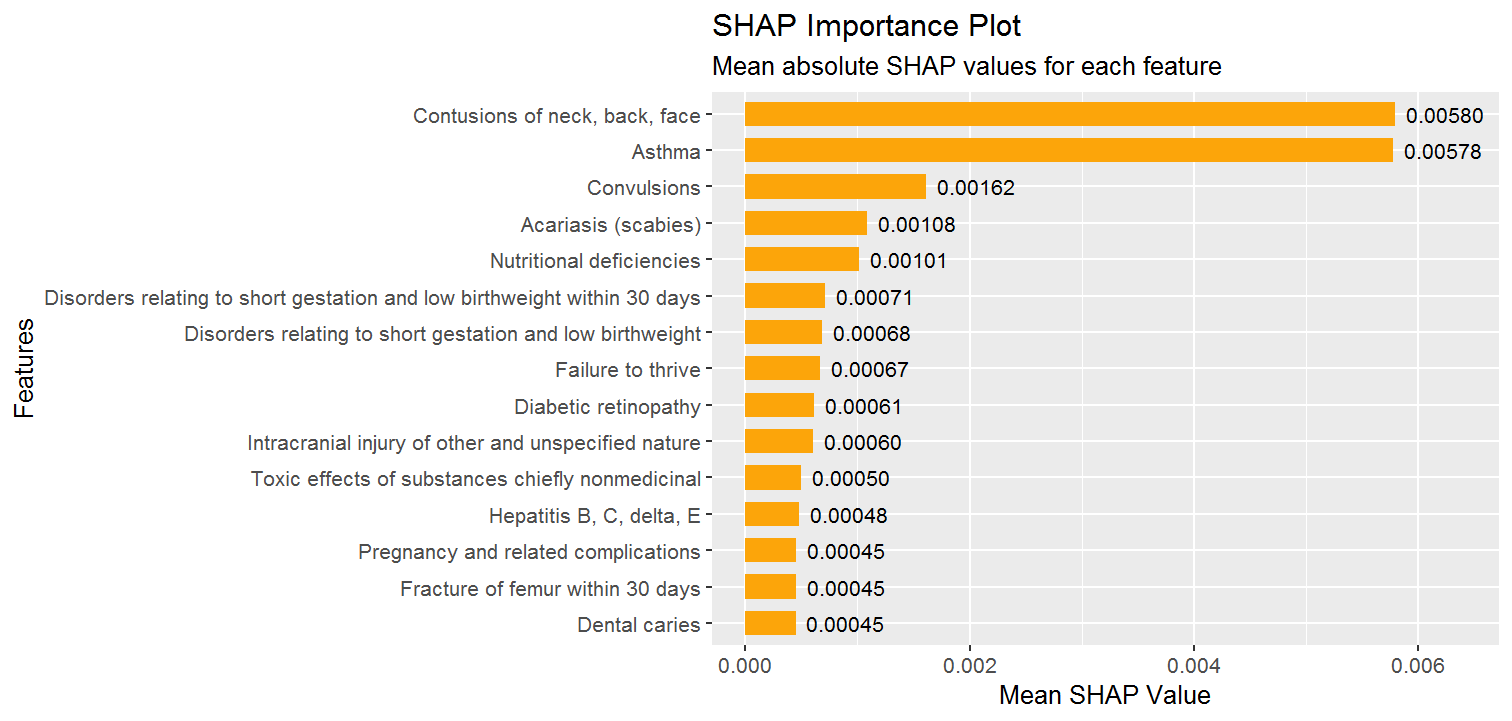

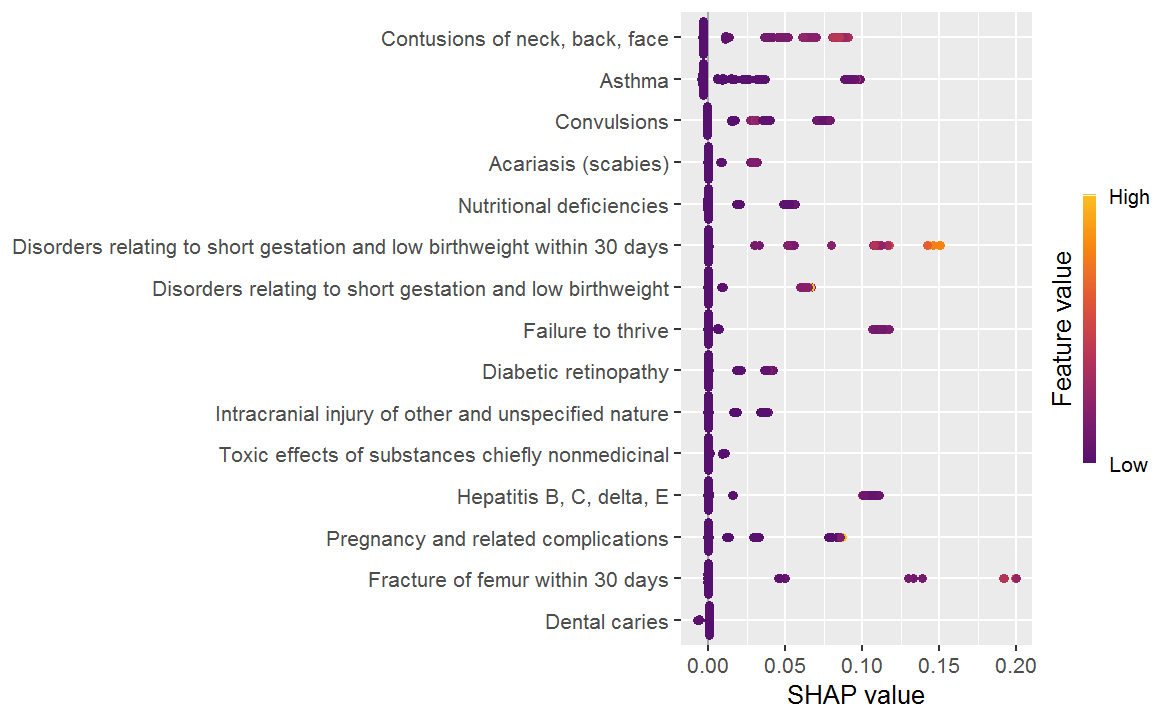


References

1. Schnitzer PG, Slusher PL, Kruse RL, Tarleton MM. Identification of ICD codes suggestive of child maltreatment. *Child Abuse Negl*. 2011;35(1):3-17. doi:10.1016/j.chiabu.2010.06.008
